# Association of Environmental Features and the Risk of Alzheimer’s Dementia in Older Adults: A Nationwide Longitudinal Case-Control Study

**DOI:** 10.3390/ijerph16162828

**Published:** 2019-08-08

**Authors:** Chih-Ching Liu, Chung-Yi Li, Shiann-Far Kung, Hsien-Wen Kuo, Nuan-Ching Huang, Yu Sun, Susan C. Hu

**Affiliations:** 1 Department of Public Health, College of Medicine, National Cheng Kung University, Tainan 701, Taiwan; 2 Department of Public Health, College of Public Health, China Medical University, Taichung 404, Taiwan; 3 Department of Urban Planning, National Cheng Kung University, Tainan 701, Taiwan; 4 Healthy Cities Research Center, Research and Services Headquarters, National Cheng Kung University, Tainan 701, Taiwan; 5 Institute of Environmental and Occupational Health Sciences, National Yang-Ming University, Taipei 112, Taiwan; 6 Department of Neurology, En Chu Kong Hospital, New Taipei City 23702, Taiwan

**Keywords:** physical environments, social environments, Alzheimer’s dementia, incidence, case-control study, longitudinal study

## Abstract

Little is known about the association between environmental features and the risk of Alzheimer’s dementia (AD). This study aims to investigate the association of physical and social environments with the incidence of AD. We identified 12,401 newly diagnosed AD cases aged ≥65 years in 2010, with the same no. of matched controls from National Health Insurance claims in Taiwan. Environmental data were collected from government statistics including three physical environments and three social environments. Multilevel logistic regression was conducted to calculate the odds ratios (OR) of AD in association with environmental features at the township level. Results showed that living in the areas with higher availability of playgrounds and sport venues was associated with a 3% decreased odds of AD (95% CI = 0.96–0.99), while higher density of elderly living alone was associated with a 5% increased odds of AD (95% CI = 1.01–1.11), after controlling for individual and other environmental factors. In further examination by urbanization level, the above relationships were found only in rural areas but not in urban areas. This study provides evidence that specific physical and social environmental features have different impacts on the risk of AD.

## 1. Introduction

Alzheimer’s dementia (AD), accounting for approximately 50–70% of overall dementia in the world, has been noted as an increasingly serious health problem [1]. As indicated by previous research, AD is a multifactorial disease [1,2]. Numerous studies have reported a range of individual factors such as smoking, lack of physical activities, lack of social engagement, and chronic disease (vascular disease, diabetes mellitus, and depression) would contribute to the risk of AD [1,2]. Nevertheless, environmental factors, such as social interaction and physical activity, might also be related to the risk of AD [3,4,5] and other health outcomes (vascular disease, diabetes mellitus, and depression) [6,7,8].

Among the limited number of published paper, a few community-based studies focusing on community resources [5,9,10], public open spaces [9,10], natural environments [10,11,12], neighborhood social cohesion [13], and socioeconomic composition of residential population [14,15,16] have found significant links between environmental characteristics and cognitive function in later life, probably through cognition stimulation [3] and cognition reserve [17]. However, most of the above-mentioned studies were cross-sectional in design, which makes it hard to explain the causal relationship between environmental characteristics and cognitive function [10,11,13,14,15,16]. Whether the environmental factors could really affect or prevent the development from AD has seldom been well-investigated. Thus, this study aims to explore the relationship between environmental characteristics and the risk of AD by using a nationwide longitudinal case-control study design and seeks to find the most associated environmental features.

## 2. Materials and Methods

### 2.1. Study Design and Data Sources

This is a nationwide population-based case-control study with a retrospectively longitudinal follow-up period from 2003 to 2011. Data were retrieved from three national datasets, including (1) Taiwan’s National Health Insurance Research Data (NHIRD), (2) the Age-Friendly Environment Database, and (3) the 2006 National Land Use Investigation. 

Individual data were collected from three parts of the NHIRD, as provided by the National Health Insurance Administration (NHIA), Ministry of Health and Welfare, Taiwan. These parts included ambulatory care claims, all inpatient claims and the updated registry for beneficiaries. The completeness and accuracy of the NHIRD has been documented in prior studies [18]. In brief, the NHIRD provides all ambulatory care and inpatient claims, and the updated registry for beneficiaries covers around 99% of Taiwanese people [19]. The NHIA performs expert reviews quarterly on a random sample of 50–100 ambulatory care and inpatient claims to ensure the accuracy of the claim files [19]. Access to the NHIRD was approved by the National Health Research Institutes Review Committee. Approved code by the National Health Research Institutes Review Committee: NHIRD-101-565. Personal identification numbers in the NHIRD are encrypted.

Ecological information on the features of physical and social environments was obtained from the Taiwan Age-Friendly Environment Database at the township level, provided by Hu et al [20]. Data on the types of land use, including (1) parks, greeneries, and square area, and (2) playgrounds and sport venues, were derived from the 2006 National Land Use Investigation conducted by the National Geographic Information System in Taiwan (https://ngis.nat.gov.tw/en/index.html).

### 2.2. Identification for Cases and Controls

We recruited incident cases of AD (ICD-9 CM codes of 290.0, 290.1, 290.2, 290.3, or 331.0) aged 65 years and older in 2010, for these ICD-9 codes are most commonly applied as clinically diagnostic codes for AD by neurologists. This definition was also adapted from some previous studies on AD which used Taiwan’s NHIRD [21,22,23]. To elevate the specificity of identifying AD cases, we selected our incident AD subjects based on the following criteria: (1) having at least three outpatient claims of these dementia-related diagnosis codes after the first AD diagnosis, and (2) the first and last outpatient visits within our study period (i.e., 2010–2011) should be at least 90 days apart. Additionally, we excluded those who had medical claims (either ambulatory or inpatient care) with diagnostic codes of stroke (ICD-9-CM code: 430–438), drug-induced mental disorders (ICD-9-CM code: 292), alcohol-induced mental disorders (ICD-9-CM code: 291), and Parkinson’s disease (ICD-9-CM code: 332) prior to their first AD diagnosis.

To identify the earliest possible date of AD diagnosis, we traced their medical history back to 2003 and excluded those who had any prior dementia-related diagnoses (from 2003 to 2009) to ensure all the subjects in the case group were incident AD cases diagnosed in 2010. The date that the AD diagnosis was first made was defined as the index date. Thus, those patients who have moved to institutions before the index date were excluded because they might have had various interactions with their living environments, and those without information on living area in 2006 were also excluded. Finally, we identified 12,401 eligible AD cases.

**Figure 1:**
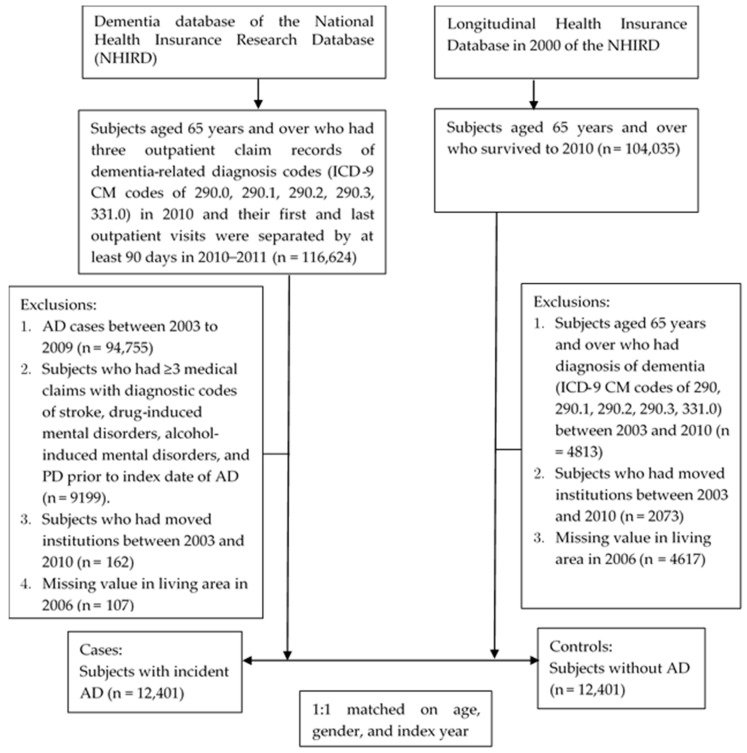
Flow chart of the enrollment in the case and control groups of Alzheimer’s dementia.

For each case, we applied a random sampling method to select one sex-, age- and index-year matched control from the Longitudinal Health Insurance Database in 2000 of the NHIRD registry. The medical history of all control individuals were also traced back to 2003. Those with the following conditions were excluded: (1) diagnosis of dementia (ICD-9 CM codes of 290.0, 290.1, 290.2, 290.3, or 331.0) between 2003 and 2010, (2) moved institutions from 2003 to 2010, and (3) missing information on living area (see Figure 1).

We identified the environment of our study subjects by using their township. Each study subject’s township was obtained from the updated registry for beneficiaries of Taiwan’s NHIRD. In Taiwan, townships were designed as administrative areas, under the counties or cities. The average size of townships was 98.33 km^2^ and the average household of township was 20,094 in the study year [24]. Zip codes were set based on township locations in Taiwan and the size of each township/zip code was determined based on population density. Besides, the average population of townships was 62,164 (0.1–41.25 thousand people /per km^2^) in 2006 [24]. Although government usually used townships as basic units to plan health-care resource or health-related policies, little study had overcome the geographic difficulty to use township as basic unit to explore the effects of environmental features on the risk of AD in older adults.

### 2.3. Measurement for Physical and Social Environments

First, we selected some physical and social environments as potentially analytical variables based on previous research [5,9,10,11,12,13,14,15,16] and available datasets in Taiwan. Since little is known about which environmental features are related to AD risk [5], we determined the environmental indicators through several meetings by our research team and experts until a consensus was reached. Finally, three physical environments and three social environments at the township level were selected in the analysis.

The three physical environments at the township level included in our study were: (1) parks, greeneries, and square area, (2) playgrounds and sport venues, and (3) community centers. We used the density of parks, greeneries, and space (in km^2^/ per 10^5^ people) as a proxy for the availability of green environment. The density of playgrounds and sport venues (in km^2^/ per 10^5^ people) were also evaluated as a proxy for the availability of recreational resources. The above types of land use were calculated from the 2006 National Land Use Investigation using ArcGIS analysis (ArcMap, version 10.3; ESRI Inc., Redlands, CA, USA). The unit of area within a township was computed as per capita area (km^2^). Lastly, the presence of community centers (as a proxy for the availability of community resources) was calculated by averaging the number of community centers for all the people of that particular township, which was derived from government statistics.

For the social environments at the township level, we included the following three features: (1) median annual family income, (2) percentage of illiterate people aged ≥65, and (3) density of elderly living alone (number/per 10^3^ elderly people) which were obtained from the 2006 government statistics, corresponding to the year of land use investigation. “Median annual family income” and “percentage of illiterate people aged ≥65” were used to represent the socioeconomic and educational level in each township where subjects lived, respectively. Also, we hypothesized that living in areas with a higher density of elderly living alone may have limited social cohesion, increased social isolation [25], and reduced social support [26].

### 2.4. Levels of Urbanization

Levels of urbanization were measured based on the following four indicators: The number of residents, the percentage of people working in secondary industries, the percentage of people working in tertiary industries, and population density using the method by Huang et al. [27] where they categorized the levels of urbanization into five levels ranging from 1 (lowest) to 5 (highest). To maintain enough statistical power, levels of township urbanization in our study were re-categorized from five levels into three levels: Urban (level 5), suburban (level 4 and level 3), and rural (level 2 and level 1).

### 2.5. Potential Confounders

Since the accessibility of medical resources may influence the diagnostic rate of AD, we adjusted for the density of hospitals and clinics (no. of hospitals and clinics per 1000 elderly people) in each township, which was retrieved from the published open data from the Taiwan Medical Association (http://www.tma.tw/stats/index_AllPDF.asp).

Other adjusted individual confounders were the followings: Occupation status (white collar, blue collar, and others), salary-based insurance premium (no salary, 0–19,200 NT$, and ≥19,200 NT$) in 2006, and no. of comorbidities including: Hypertension, diabetes, heart disease, head injury, hyperlipidemia, depression, and chronic obstructive pulmonary disease. The number of comorbidities from 2003 to 2005 was computed when subjects had ≥3 of these comorbidities within 1 year in outpatient claims or one comorbidity in inpatient claims.

### 2.6. Statistical Analysis

**Figure 2:**
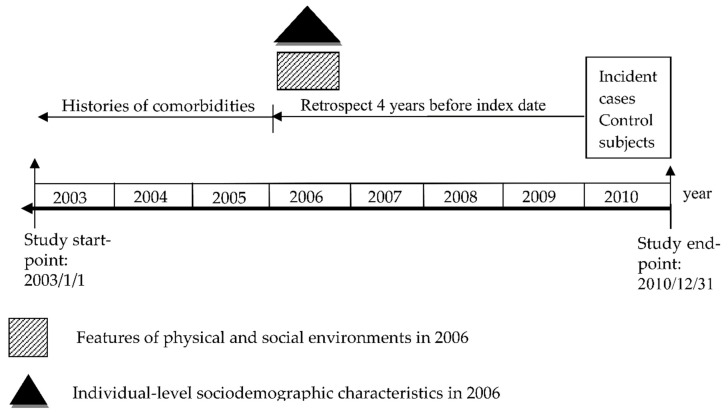
Exposure time for the environmental features in the case and control groups.

We used a diagram to illustrate the exposure time of physical and social environments or covariates in cases and controls (see Figure 2). Pearson’s χ^2^ test was first performed to compare individual characteristics between AD cases and controls. Next, minimum, maximum, mean ± standard deviation, quartiles, and interquartile range increase (IQR) of physical and social environment data in the living township of the study subjects in 2006 were described. Because the environmental characteristics were not normally distributed in this study, the environmental measures were dichotomously characterized (i.e., <median and ≥median) and then the differences in environmental characteristics were compared between cases and controls by Pearson’s χ2 test.

We subsequently performed two-level random intercept logistic regression models to investigate the odds ratios (ORs) and 95% confidence intervals (CI) of AD risk in relation to township-level features of physical and social environments. ORs were described for an IQR increase. We adapted the GLIMMIX procedure in SAS (version 9.4, SAS Institute Inc., Cary, NC, USA) to fit multilevel logistic regression models (individual at level 1 nested within townships/cities at level 2) with a binary outcome and a logit link [28]. To detect multicollinearity, variance inflation factor (VIF) was examined among the physical and social environment factors and covariates. All VIF in the regression model was less than 3, which is good for testing a model.

To assess the difference of AD risk across townships, we first used the null model (i.e., no predictors were included in the model) to examine the error variance level-2 intercept (Model 0). Second, we added the characteristics of the physical environment at the township level into the model to examine their association with AD risk (Model 1). Third, the characteristics of social environment at the township were also entered into model to predict the AD risk (Model 2). Fourth, the characteristics of the physical and social environment were entered into the model simultaneously before adjustment (Model 3) and after adjustment for individual-level factors (occupation, salary-based insurance premium, and number of comorbidities) (Model 4). Lastly, further adjustments for the density of hospitals/clinics and levels of urbanization were conducted in Model 5 (full model) to examine the degree to which the relationship between the physical and social environment characteristics and the AD risk changed. A *p*-value <0.05 was considered statistically significant.

## 3. Results

The cases and controls were comparable regarding gender and age. AD cases were less likely to be white-collar workers (11.9% vs. 12.5%), less likely to have higher insurance premium (% with ≥median insurance premium: 37.3% vs. 39.9%), and more likely to have ≥3 comorbidities (19.2% vs. 17.1%) compared with in the control group (Table 1). The features of physical and social environments in the 346 townships of the study in 2006 were highly skewed (Table 2). Among these townships, cases and controls were nested within the 337 and 338 townships, respectively. Table 3 shows that case and control subjects were similar in regard to the density of parks/greeneries, density of playgrounds and sport venues, percentage of illiterate people aged ≥65, and density of elderly living alone, but were significantly different in the density of community centers (≥median: 18.0% vs. 19.0%), median annual family income (≥median: 79.7% vs. 78.6%), density of hospitals and clinics (≥median: 84.1% vs. 82.9%), and urbanization level (urban: 27.3% vs. 27.0%; suburban: 47.9% vs. 46.1%).

Results of multilevel logistic regression for the association between environmental features and the risk of AD are shown in Table 4 and Table 5. In Table 4, the null model suggests there is significant variability in AD incidence between townships in case-control samples (error variance level-2 intercept: 0.03198, *p* < 0.0001). For physical environments, we observed that per IQR increases in playgrounds, sport venues, and community centers were inversely associated with the risk of AD, with an adjusted OR (AOR) of 0.97 (95% CI = 0.96–0.99) and 0.92 (95% CI = 0.86–0.99), respectively (Model 1). For social environments, the adjusted OR of AD was 1.05 (95% CI = 1.01–1.10) for an IQR increase in median annual family income in the living township (Model 2).

After adjustment for both physical and social environment factors, playgrounds/sport venues, and community centers still had significant effects on the risk of AD (Model 3). The relationship between per IQR increase in elderly living alone and the risk of AD was significant (AOR = 1.05, 95% CI = 1.01–1.10) after adjustment of physical and social environment characteristics. When we further adjusted for individual factors (insurance premium, occupational status, and number of comorbidities), the IQR increase in playgrounds/sport venues and elderly living alone still showed a significant association with AD with the adjusted OR at 0.97 (95% CI = 0.96–0.99) and 1.05 (95% CI = 1.01–1.11), respectively (Model 4). In Model 5 with further adjustment for “density of hospitals and clinics” and “urbanization status” based on Model 4, per IQR increases in playgrounds/sport venues (AOR = 0.97, 95% CI = 0.96–0.99) and elderly living alone (AOR = 1.05, 95% CI = 1.01–1.11) remained significantly associated with the risk of AD.

In Table 5, we found that urbanization level may change the effects of specific features of environments on the risk of AD. For subjects living in urban areas, physical and social environments had no significant effect on the risk of AD. However, in suburban areas, per IQR increase in illiterate elderly aged ≥65 was positively associated with the risk of AD (adjusted OR = 1.24, 95% CI = 1.04–1.48). For elderly living in rural areas, per IQR increase in playgrounds/sport venues or community centers was inversely associated with the risk of AD, which meant there was a protective effect with an adjusted OR of 0.97 (95% CI = 0.94–0.99) and 0.89 (95% CI = 0.79–0.99), respectively. Meanwhile, the adjusted OR of AD associated with per IQR increase in elderly living alone was 1.11 (95% CI = 1.03–1.20) in rural areas.

## 4. Discussion

### 4.1. Main Findings

This study is distinguishable from previous works by its nationwide scope and longitudinal study design, which aims to compare the environmental features of townships with the risk of developing AD from an overall aspect or by urbanization level. In this large-scale case-control study, we found that older adults living in areas with higher density of playgrounds and sport venues were independently associated with a 3% decreased odds of AD. Also, those living in the areas with higher density of elderly living alone may have a 5% increased risk of AD, after adjustment for individual and other confounding factors. Further examining the effects of urbanization levels, we found that physical and social environments had no significant effect on the risk of AD for subjects living in urban areas; environmental effects were found only in rural and suburban areas. Namely, in rural areas, high density of playgrounds/sport venues and community centers showed a protective effect with a 3% and 11% decreased odds of AD, respectively, whereas a high density of elderly living alone showed an adverse effect with an 11% increased odds. Yet, in suburban areas, only a higher density of illiterate elderly aged ≥65 was found to be related to an adverse effect (24% increased odds of AD).

### 4.2. Physical and Social Environments and AD in Later Life

Our data showed the adjusted OR of AD was 0.97 (95% CI = 0.96–0.99) per IQR increase in playgrounds and sport venues in any adjusted models, which is similar to the results from a previous UK study [11]. This cross-sectional UK study found nearly 60% decreased odds (95% CI = 0.23–0.82) of dementia in older adults living in areas with greater mixed land use (inclusion of residential, commercial and recreational facilities, services, and resources) after controlling for individual factors and area deprivation [11]. The mechanism by which factors may mediate the relationship between the availability of playgrounds/sport venues and AD development has not been identified. One possible explanation is that people living in areas with increased availability of places for physical activity may have more leisure-time for physical activities [29] and social interactions [30]. Physical activity, exercise, and social network are all contributors to the build-up of cognition reserve [17] and enhancing mental health [31,32] to prevent or delay the development of AD [1,2].

Noticeably, our study found an interesting environmental feature where more elderly living alone (as a proxy for lower social cohesion in local areas) is an independent significant factor associated with the risk of AD (OR = 1.05, 95% CI = 1.01–1.11). The mechanism of how this factor may affect AD remains unclear. It has been suggested that individuals living in areas that are less socially cohesive are more likely to have secondary behavior [33], to smoke [33], to have depression [33], hypertension [34], myocardial infarction [35], and to have lower cognitive skills and abilities in older adults [13], thus increasing the risk of AD development. 

Although the only published longitudinal cohort study through an 18-year follow-up period suggested that older people living in a neighborhood with higher community resources had slower rates of cognitive decline independent of individual factors [9], our study found the protective effect of living in areas with higher community centers on the risk of AD was no longer significant after adjustment of individual factors. This meant the effect of community centers on the risk of AD was largely due to individual factors of the older adults living in these areas. Our study showed that more white- and blue-collar workers (75.9% vs. 45.2%), older people with lower dependence (19.3% vs. 41.5%), and less comorbidities (≥ three: 15.5% vs. 18.8%) tended to live in townships with a high community center density rather than in townships with a low community center density (data not shown). Thus, it is concluded that the protective effect of community centers decreased due to those individual factors. We suspected that people with those individual factors or have higher educational level would have better self-selection to live in areas with highly facilitated community center because they can obtain more community resources in that area. Much more work in the future is needed be done to understand how individual education and community center interrelate and influence each other.

### 4.3. Effects of the Urbanization Levels

Urbanization may affect the association between environmental factors and dementia risk. A previous study from the UK found that high availability of green environments would increase the odds of cognitive impairment in rural areas (OR = 1.30, 95% CI = 1.09–1.57), which might indicate that a lack of environmental stimulation could be harmful for cognitive performance [10]. In contrast, high availability of green environments in conurbations might provide a “psychological-restoration” setting, buffer against stress [36,37], and reduce stimulation overload [3], which could result in a positive effect on cognitive health [10]. In this study, we assessed the area of greenery and found these factors had no urban–rural differences with relation to the risk of AD. Whether this finding can be generalized for the availability of green environment in smaller areas in Taiwan needs further investigation.

Previous studies have also shown that the association between neighborhood mixed land use and cognitive health was modified by levels of urbanization [10,38]. Similar to the previous studies, our study showed that increased availability of “playgrounds and sport venues” and “community centers” has significantly protective effects on the risk of AD onset particularly in rural areas. Some reasons could explain our findings. First, the effects of environmental characteristics in a small area on cognitive health can vary largely across levels of urbanization [3,10,38], which may result from different interactions of residents with their urban or rural environments [39]. For example, urban areas usually have many forms of transportation systems available. People in rural areas, however, have less access to transportations and are stuck on their confined environment. Hence, the impact of the immediate environment may be more relevant for people in rural areas. A prior study showed older adults in rural areas have inadequate resources or limited connections for leisure and physical activity, which may contribute to a decreased level of physical activity [27] and lack of environmental stimulation [3]. The above conditions may also contribute to individual cognitive impairments and in turn to increase the risk of dementia in later life [1,3]. Thus, older people living in areas with higher availability of playgrounds/sport is considered beneficial for cognitive health and in delaying or preventing AD development. Second, rural areas might be related to social isolation, loneliness, restricted opportunities for social interaction [32,40], which could weaken cognitive function and thus increase the risk of dementia in older adults [13,41]. Playgrounds/sport are venues and community centers acting as a supportive environment to encourage social participation [30] can provide significant protection effects on cognition and then compensate the impact of the limited social interaction [41].

Similarly, we found higher densities of elderly living alone significantly increased the risk of AD onset in rural areas but not in urban and suburban areas. This might be due to the fact that older residents living in rural areas with higher densities of elderly living alone might keep them from having better social cohesion [25,40] and induce feelings of loneliness [32], which lead to negative influences on cognitive health [13] and increase the risk of dementia [41].

Additionally, we also found that the association of “density of illiterate people aged ≥65” at the township-level with the risk of developing AD was modified by levels of urbanization. Although areas with per IQR increase in illiterate elderly were not associated with the risk of developing AD overall, this feature was significantly associated with a 24% increased adjusted odds of AD in suburban areas after controlling for individual and other environmental factors. In Taiwan, suburban residents suffer from greater stress, with a potentially negative influence on mental health [42] that may increase the risk of developing dementia [43]. But the reason why people living in suburban areas are affected by environments with higher rates of illiteracy in terms of AD risk is still unclear and needs to be further explored.

### 4.4. Strengths and Limitations

Several strengths were noted in this study. First, to the best of our knowledge, this study is the first to explore the role of both physical and social environmental features overall and across levels of urbanization in determining the risk of developing AD in later life. We used a large and representative sample from the NHI database, yielding little chance of selection bias and ample sample size of urbanization-disperse patients. In addition, all computerized information on environmental characteristics would limit the subject’s recall bias. Second, by using the case-control design with longitudinal follow-up, we collected exposure information more efficiently. All exposure information collected before the new diagnosis of AD was beneficial for a causal interpretation of the results. Third, we used diagnosed cases with AD instead of prevalent cases with AD which makes the possibility of prevalence–incidence bias less likely.

Despite these advantages, our study also has several limitations. First, we only selected cases with AD based on the physician-recorded diagnosis from NHI datasets, which might have caused disease misclassification of AD. To address this concern, we included solely AD cases diagnosed with ≥3 ambulatory visits and their first and last outpatient visits were separated by at least 90 days to decrease the likelihood of disease misclassification. Second, given the accessibility and availability of medical care varied across urbanization levels [44,45], the patients with AD living in different levels of urbanization may have different opportunities to be diagnosed as having AD. To deal with this problem, the density of hospitals and clinics in each township was adjusted as a covariate to decrease this potential confounding effect. Third, we did not adjust for all potential confounders related to AD, including smoking, educational level, occupation, physical function, social participation, genes, and specific environmental features like neighborhood psychosocial disorders (e.g., crime), public transport availability, and pollutants, because information on these variables were lacking in the database. Although we used occupational status and COPD as surrogates for individual education level and COPD (i.e., one of the comorbidities), respectively, it is possible that there was residual confounding bias in our study. Fourth, the exposure time in this study was not long enough. The pathologies of AD, including amyloid deposition, neurofibrillary tangles, progressive loss of synapses and neurons, may start 10–20 years before the onset of AD symptoms [46]. Because the earliest official version of precise nationwide land use data in Taiwan became available in 2006, we had no choice but to base our measures of environmental characteristics on the data in 2006, which may not accurately represent long-term exposures in relation to the risk of developing AD. Due to limited availability of land-use data, we were unable to measure the exposures to physical and social environment features at different times throughout the course of life, which might yield some degrees of environmental exposure misclassification in our study. We assume the exposure misclassification would be similar between the case and matched control groups. Fifth, in the analysis of residential mobility among our study subjects, we found that nearly 40% of them lived in different residential areas 5 years prior to the year 2006. This may result in potential relocation bias in our study. Last, in Taiwan, physicians diagnosed Alzheimer’s Disease according to patient’s medical records, laboratory data, cognitive tests, and brain images to exclude other diseases with Alzheimer’s Disease -like symptoms. However, in this study all AD cases were selected based on clinical diagnosis made by physicians. Whether or not biomarkers of Alzheimer’s disease were used for AD diagnosis by clinicians was unknown. It is possible that other kinds of degenerative-type dementia like frontal lobe dementia may have been misdiagnosed as AD. Evidence shows that many clinically diagnosed Alzheimer’s disease was actually AD mixed with vascular pathology or other pathologies [47]. Lack of information on imaging or biomarkers is also a major limitation of this study.

## 5. Conclusions

In conclusion, living in townships with high availability of playgrounds and sport venues may reduce the risk of developing AD, while living in areas with high density of elderly living alone has the opposite effect. Additionally, the effects of specific physical and social environmental features on AD incidence were different according to urbanization status. In rural areas, older adults may significantly benefit from living in areas with higher availability of playgrounds/venues and community centers, but a high risk of AD was shown in areas with a higher density of elderly living alone. In suburban areas, older residents living in areas with high illiteracy may have higher risk of developing AD.

In light of this, we provide a few suggestions for policy implementation. First, since higher density of elderly living alone and lower availability of playgrounds/sport venues has negative effects on elder’s cognition, we suggest identify these townships firstly and improve the above-mentioned situations appropriately may be a viable strategy to prevent or reduce the risk of AD at population level. Second, future public health interventions should consider the rural-urban differences in environmental features and therefore develop appropriate strategies for preventing or delaying AD occurrence according to urban and rural features.

## Figures and Tables

**Table 1:** Characteristics of participants according to Alzheimer’s dementia.

Variables ^a^	Case		Control		*p* ^e^
	n	%	n	%	
Gender					1.000
Men	5071	40.9	5071	40.9	
Female	7330	59.1	7330	59.1	
Age (years)					1.000
65–69	1135	9.2	1135	9.2	
70–74	2132	17.2	2132	17.2	
75–79	2692	21.7	2692	21.7	
80–84	3215	25.9	3215	25.9	
≥80	3227	26.0	3227	26.0	
Mean ± SD ^b^	79.6 ± 7.2		79.3 ± 7.1		
Occupational status					<0.0001
White collar	1479	11.9	1549	12.5	
Blue collar	4608	37.1	4984	40.2	
Others	6314	51.0	5868	47.3	
Salary-based insurance premium (NTD) ^b^					
dependents	4592	37.4	4622	37.2	<0.0001
<Median (19,200)	3093	25.3	2829	22.8	
≥Median	4578	37.3	4950	39.9	
Mean ± SD ^b,c^	8139.8 ± 11,021.6		8718.7 ± 11,517.4		
Number of comorbidities ^d^					0.0011
0	4034	32.5	4181	33.7	
1–2	5985	48.3	6096	49.2	
≥3	2382	19.2	2124	17.1	
Total	12,401	100.0	12,401	100.0	

^a^ Inconsistency between total population and population summed for individual variables due to missing information. ^b^ SD = Standard deviation; NTD = New Taiwan Dollars. ^c^ The dependent insurers were not included. ^d^ Number of comorbidities included hypertension, diabetes, heart disease (i.e., coronary artery disease or congestive heart failure), head injury, hyperlipidemia, depression, and chronic obstructive pulmonary disease. ^e^ Based on χ^2^ tests.

**Table 2:** Descriptive statistics of environmental features in the residential township (n = 346) of the study subjects.

Environmental Features	Percentile						
	Min	Max	Mean ± SD ^a^	Q1^a^	Q2 ^a^	Q3 ^a^	IQR ^a^
**Physical Environments**							
Parks, greeneries, and square area ^b^	0	25.98	0.54 ± 1.50	0.14	0.29	0.55	0.41
Playgrounds and sport venues ^b^	0	17.76	0.97 ± 2.15	0.08	0.21	0.80	0.72
Community centers ^c^	0	324.75	41.95 ± 43.49	10.11	31.17	59.42	49.31
**Social environments**							
Median annual family income ^d^	339	829	507.46 ± 68.16	465	494	539	74
Illiterate people aged ≥65 ^e^	0.22	55.93	18.79 ± 11.03	10.08	16.74	26.73	16.65
Elderly living alone ^f^	0	539.29	38.41 ± 51.27	10.85	18.89	46.46	35.61
**Other environments**							
Hospitals and clinics ^f^	0.72	33.82	6.43 ± 4.99	2.74	5.00	8.72	5.98

^a^ SD = Standard deviation; Q1 = 25 percentile; Q2 = 50 percentile (Median); Q3 = 75 percentile; IQR = Interquartile range. ^b^ km^2^/ per 10^5^ people. ^c^ number/per 10^5^ people. ^d^ 1000 New Taiwan Dollars. ^e^ %. ^f^ number/per 10^3^ elderly people.

**Table 3:** Environmental features of township level in Alzheimer’s dementia cases and controls.

Variables	No. of Township (Case/Control)	Case		Control		*p* ^f^
		n	%	n	%	
**Total**	**337/338**	12,401	100.0	12,401	100.0	
**Physical environments**						
Parks, greeneries, and square area ^a^						0.6843
<Median	172/172	6434	51.9	6402	51.6	
≥Median	165/166	5967	48.1	5999	48.4	
Playgrounds and sport venues ^a^						0.0628
<Median	170/169	8351	67.3	8213	66.2	
≥Median	167/169	4050	32.7	4188	33.8	
Community centers ^b^						**0.0499**
<Median	173/170	10,162	82.0	10,042	81.0	
≥Median	164/168	2239	18.0	2359	19.0	
**Social environments**						
Median annual family income ^c^						**0.0335**
<Median	163/166	2518	20.3	2654	21.4	
≥Median	174/172	9883	79.7	9747	78.6	
Illiterate people aged ≥65^d^						0.7144
<Median	165/168	8381	67.6	8354	67.4	
≥Median	172/170	4020	32.4	4047	32.6	
Elderly living alone ^e^						0.7760
<Median	173/170	8373	67.5	8352	67.4	
≥Median	164/168	4028	32.5	4049	32.6	
**Other environments**						
Hospitals and clinics ^e^						**0.0080**
<Median	168/167	1971	15.9	2126	17.1	
≥Median	169/171	10,430	84.1	10,275	82.9	
Urbanization						**0.0005**
Rural	196/199	3076	24.8	3338	26.9	
Suburban	113/111	5937	47.9	5714	46.1	
Urban	28/28	3388	27.3	3349	27.0	

^a^ km^2^/per people. ^b^ number/per 10^5^ people. ^c^ 1000 New Taiwan Dollars. ^d^ %. ^e^number/per 10^3^ elderly people. ^f^ Based on χ^2^ tests.

**Table 4:** Odds ratio of Alzheimer’s dementia in relation to individual characteristics and environmental features^#^ in the residential township of the study subjects.

Variables	Model 1	Model 2	Model 3	Model 4	Model 5
**Intercept (SD) ^a^**	0.02 (0.03)	−0.45 (0.19) *	−0.21 (0.20)	−0.16 (0.21)	−0.20 (0.21)
**Physical environments**					
Parks, greeneries, and square area	1.02 (0.99–1.05)		1.02 (0.99–1.05)	1.02 (0.99–1.05)	1.01 (0.99–1.04)
Playgrounds and sport venues	0.97 (0.96–0.99) *		0.97 (0.96–0.99) *	0.97 (0.96–0.99) *	0.97 (0.96–0.99) *
Community centers	0.92 (0.86–0.99) *		0.91 (0.83–0.98) *	0.92 (0.85–1.01)	0.94 (0.86–1.03)
**Social environments**					
Median annual family income		1.05 (1.01–1.10) *	1.02 (0.98–1.07)	1.01 (0.96–1.06)	1.01 (0.96–1.05)
Illiterate aged ≥65		1.04 (0.97–1.12)	1.04 (0.97–1.12)	1.06 (0.99–1.14)	1.08 (0.99–1.16)
Elderly living alone		1.03 (0.99–1.08)	1.05 (1.01–1.10) *	1.05 (1.01–1.11) *	1.05 (1.01–1.11) *

In the null model, the estimated error variance level-2 Intercept (SD) was also 0.03198 (0.008055), *p* < 0.0001. ^#^ Environmental exposure as 1 IQR. ^a^ SD = Standard deviation. Model 1: Physical environment factors only. Model 2: Social environment factors only. Model 3: Model 1+ Model 2. Model 4: Model 3 + individual variables (occupational status, insurance premium, and no. of comorbidities). Model 5: Model 4 + hospitals and clinics, and urbanization status. * *p* < 0.05.

**Table 5:** Odds ratio of Alzheimer’s dementia in relation to environmental features^#^ and individual characteristics in the residential township by level of urbanization.

Variables	Urban areas ^b^	Suburban Areas ^b^	Rural Areas ^b^
**Intercept (SD) ^a^**	−0.08 (0.65)	−0.65 (0.37)	0.28 (0.45)
**Physical environments**			
Parks, greeneries, and square area	0.93 (0.76–1.14)	0.99 (0.93–1.06)	1.03 (0.99–1.07)
Playgrounds and sport venues	1.24 (0.22–6.87)	0.97 (0.95–1.00)	0.97 (0.94–0.99) *
Community centers	0.78 (0.42–1.44)	0.96 (0.81–1.14)	0.89 (0.79–0.99) *
**Social environments**			
Median annual family income	1.01 (0.91–1.12)	1.05 (0.97–1.14)	0.95 (0.84–1.06)
Illiterate people aged ≥65	1.12 (0.63–2.00)	1.24 (1.04–1.48) *	1.03 (0.95–1.13)
Elderly living alone	1.02 (0.93–1.12)	1.06 (0.96–1.16)	1.11 (1.03–1.20) *

^#^ Environmental exposure as 1 IQR. ^a^ SD = Standard deviation. ^b^ Based on multilevel logistic regression and adjusted for insurance premium, occupational status, no. of comorbidities, parks and greeneries, playgrounds and sport venues, community center, median annual family income, illiterate people aged ≥65, elderly living alone, hospitals and clinics, and urbanization status. * *p* < 0.05
